# Surgical Approach and Variation in Long-Term Survival Following Colorectal Cancer Surgery Using Instrumental Variable Analysis

**DOI:** 10.1097/AS9.0000000000000538

**Published:** 2025-01-23

**Authors:** Cody Lendon Mullens, Sarah Sheskey, Edward C. Norton, Jyothi R. Thumma, Hari Nathan, Scott E. Regenbogen, Kyle H. Sheetz

**Affiliations:** From the * Department of Surgery, University of Michigan, Ann Arbor, MI; † Center for Healthcare Outcomes and Policy, University of Michigan, Ann Arbor, MI; ‡ UM National Clinician Scholars Program, Ann Arbor, MI; § Department of Health Management and Policy, University of Michigan, Ann Arbor, MI; ‖ Department of Economics, University of Michigan, Ann Arbor, MI.

**Keywords:** colon cancer, colorectal surgery, long-term survival, rectal cancer, robotic surgery

## Abstract

**Objective:**

The study aimed to determine whether increased use of minimally invasive surgical approaches, compared with open, improves long-term survival after colon and rectal cancer resections.

**Background:**

Existing prospective and observational data comparing surgical approach for colon and rectal cancer are limited by selection bias, necessitating better approaches for causal inference to understand the relationship between surgical approach and long-term survival.

**Methods:**

We included colon and rectal cancer patients who underwent colon or rectal resection from the American College of Surgeons National Cancer Database between 2011 and 2018. Using an instrumental variable (IV) approach, we accounted for measured and unmeasured differences between patients undergoing colon or rectal cancer resection based on operative approach – robotic, laparoscopic, or open. The IV used in this study was rate of robotic-assisted colon and rectal cancer surgery within 81 different hospital regions based on US Census region and rurality during the 12 months before each patient’s operation. Proportional hazard modeling was used to estimate risk-adjusted mortality rates.

**Results:**

There were 326,406 colon and 96,979 rectal cancer patients included in this study. The risk-adjusted 5-year cumulative incidence of mortality for colon and rectal cancer was highest for patients who underwent open approaches (35.73 [95% confidence interval {CI}: 35.37–36.1] and 39.27 [95% CI: 28.44–30.13], respectively), compared with lower mortality for those undergoing laparoscopic (28.91 [95% CI: 28.55–29.27] and 22.93 [95% CI: 22.11–23.78], respectively) and robotic approaches (26.39 [95% CI: 24.51–28.42] and 19.77 [95% CI: 17.32–22.43], respectively). Growth in utilization of minimally invasive approaches outpaced improvements in long-term survival.

**Conclusions:**

Patients undergoing minimally invasive surgical approaches for colon and rectal cancer had improved long-term survival. However, long-term survival changes did not correlate with the large expansion of minimally invasive approaches, which suggests that growing these approaches is not a viable strategy to improve long-term patient outcomes.

## INTRODUCTION

In 2024, more than 150,000 people in the United States were diagnosed with colon or rectal cancer.^1^ Most patients with colorectal cancer will undergo some form of surgical resection as part of their oncologic management.^2,3^ While the COST Trial and others confirmed that laparoscopic and open resection for colon cancer are equivalent, the ALaCaRT, ROLARR, and ACOSOG Z6051 trials raised concerns about pathologic outcomes of laparoscopic and robotic versus open approaches for rectal cancer.^4–9^ Different approaches for managing colon and rectal cancer remain viable options including open surgery, while there has been substantial growth in the utilization of minimally invasive approaches (laparoscopic and robotic).^10^

While surgical approach and its implications for long-term survival have been explored using randomized and observational data, these studies are no longer contemporary and/or are plagued by selection bias. This underscores the need to leverage causal inference methodologies that minimize selection bias, to the greatest extent possible. There has been a substantial decrease in open surgical management for colorectal cancer as minimally invasive approaches have continued to grow as much as 3.5- and 41.3-fold in laparoscopic and robotic approaches, respectively, over the past 2 decades.^11^ Short-term advantages of minimally invasive approaches such as decreased postoperative pain, shorter length of stay, and less overall morbidity have been described. An additional crucial variable to consider in the management of cancer patients is long-term overall survival. However, robust assessment of long-term overall survival of colorectal cancer patients in the context of surgical approach is currently lacking. Given the prominent role of surgical management in colorectal cancer and the recent shifts in approaches for its surgical management, understanding its potential influence on long-term survival is paramount. This is especially timely given that emerging data have uncovered concerns around oncologic outcomes for robotic-assisted approaches for surgical management of breast and cervical cancer.^12–14^

In this study, we investigate differences in long-term overall survival in colorectal cancer patients based on surgical approach. To accomplish this, we used data from the American College of Surgeons (ACS) National Cancer Database (NCDB) and an instrumental variable (IV) analysis. This approach leverages geographic variation in the adoption of minimally invasive techniques over time as a natural experiment, allowing us to account for both measured and unmeasured differences between patients undergoing resection by each approach.

## METHODS

### Data Source and Study Population

We leveraged data from the ACS NCDB for patients with nonmetastatic colon or rectal cancer who underwent colon or rectal resection for cancer during the calendar years 2011 to 2018. The NCDB is a prospective hospital-based national registry that accounts for more than 70% of patients diagnosed with cancer across more than 1500 ACS Commission on Cancer accredited programs across the United States.^15^ We obtained data from NCDB on all colon and rectal cancer patients during the defined study period.

Among colon and rectal cancer surgery patients, we collected data on patient age, gender, race/ethnicity, geographic region, rurality/urbanicity, payer type, Charlson-Deyo score, clinical staging and grading information, type of cancer (eg, adenocarcinoma), and rate of emergency surgery (defined as within 3 days of diagnosis). The Charlson-Deyo score is a metric to predict the risk of death within 1 year of hospitalization based on 17 different comorbid conditions.^16^ These data were compiled separately for patients with colon cancer and patients with rectal cancer. We included patients with clinical stages 1 to 3 of colon and rectal cancer surgeries that were performed at the primary cancer site. Patients with metastatic disease (eg, stage 4) were excluded. Patients with missing follow-up data or with less than 90 days of follow-up or with missing surgery date or last contact date were excluded from the study. We also excluded patients who underwent surgery in hospital regions (described in detail below) that did not perform robotic-assisted colon or rectal cancer operations during the study period.

This study was deemed as being exempt by the institutional review board at the University of Michigan study based on its analysis of secondary data.

### Outcomes

The primary outcome of interest in this study was risk-adjusted cumulative incidence of mortality after undergoing resection for colon or rectal cancer. The NCDB data have patients’ vital status and days from diagnoses to surgery of the primary site as well as months between the date of diagnoses and last contact or death. We created follow-up time as months since surgery of the primary cancer site until death or last contact.

We identified surgical approach for colon or rectal cancer (robotic, laparoscopic, open) by a surgical approach indicator included in the NCDB data. All robotic converted to open or laparoscopic approach converted to open are categorized as open approach. Explanatory variables included in our models were done in accordance with previously published work^17^ and included patient age, sex, race/ethnicity, Charlson-Deyo score to reflect comorbidity burden, primary insurance payor, year of diagnosis, surgical approach (robotic, laparoscopic, open), clinical stage and grade, cancer type, and whether an emergent operation was performed. We defined emergent operation as the surgical procedure of the primary site being performed within 3 days from diagnosis.

### Instrumental Variable

There has been a rapid uptake of the robotic-assisted surgical approach for colon and rectal cancer surgery over the last 2 decades and is able to be differentiated from laparoscopic and open approaches in most clinical and administrative databases, including the NCDB. A recognized drawback of these data are limitations with respect to causal inference due to selection bias, which could not be accounted for with a standard logistic regression. One mitigation strategy to control for potential measured or unmeasured confounders is performing an IV analysis, which was the approach chosen here. We have used this approach to counteract similar bias in other studies.^18–20^

The IV used in this study was the rate of robotic-assisted colon and rectal cancer surgery within a hospital region in the previous 12 months to the patient’s operation. Hospital regions were defined based on 2 key measures: US census divisions and Rural-Urban Continuum Codes published by the US Department of Agriculture Economic Research Service. There are 9 US census divisions and 9 Rural-Urban Continuum Codes that measure rurality/urbanicity.^21^ Thus, combining these 2 measures, we defined 81 distinct hospital regions. We used prior-year regional-level procedural use for the 81 combinations of facility location as an IV. We excluded patients in referral regions where no robotic-assisted colon or rectal cancer operations occurred during the study period. These exclusions involved 47 colon cancer and 60 rectal cancer patients from 2 hospital regions.

### Statistical Analysis

Individual analyses as described below were done separately for colon and rectal cancer populations. Due to the nonlinear and binary outcome of mortality, a time-to-event analysis was used with a 2-stage residual inclusion estimation method for our IV model.^22^ In the first stage of analysis, multivariable logistic regression was used to estimate the likelihood of a patient undergoing robotic cancer surgery for colon or rectal cancer while adjusting for several covariates: prior-year regional use of robotic-assisted colon or rectal cancer (IV), age, sex, race/ethnicity, primary insurance payor, comorbidities, clinical stage and grade, cancer type, whether an emergent operation was performed, hospital total procedure specific surgery volume, and year of diagnosis. For the second stage, a flexible parametric survival model was created to calculate hazard ratios (HRs) and the cumulative incidence of mortality while adjusting for surgical approach, age, sex, race/ethnicity, primary insurance payor, comorbidities, clinical stage and grade, whether the operation was emergent, hospital total procedure specific surgery volume and year of diagnosis. The first and second stages of these models also accounted for clustered outcomes at the hospital region level. HRs were calculated using marginal effect estimates based on the flexible parametric survival model. Mortality rates were estimated using the same models with covariates set to their means. Proportional hazard assumptions were tested using Schoenfeld residuals. For variables that violated the assumption, we included time-varying effects option in the model. Variables requiring time-varying effects included surgical approach, age, grade, clinical stage, and emergent surgery.

To assess the strength of our IV, we first calculated the Kleibergen-Paap Wald F statistic for prior-year use of robotic-assisted colon and rectal cancer surgery and the current-year treatment. Our F statistic of 137 for the colon cancer model and 122 for the rectal cancer model demonstrated that the IVs were highly associated with undergoing robotic-assisted colon or rectal cancer. F statistic >10 is indicative of a strong instrument.^23^ A valid instrument must only be associated with the outcome through the treatment variable. While this cannot be empirically proven, it can be evaluated by examining the balance of patient characteristics when stratified based on the instrument. To accomplish this, local lagged treatment patterns are believed to satisfy this condition given that they reflect treatment decisions from a prior time period among a different set of patients. Further, regional use of robotic-assisted surgery in a prior year is likely to influence its utilization in subsequent years. For the latter stratification, we analyzed baseline patient characteristics for as-treated cohorts and for patients stratified around the median of our IV. With a strong instrument, patient covariates are ideally more similar across approaches when comparing above- and below-median levels of the instrument to the actual treatment level. We further assessed for endogeneity with a Durbin-Wu-Hausman test performed on the coefficient of the residual from the first stage of 2-stage residual inclusion estimation methods.^22^

We observed significant covariate imbalance reduction when evaluating by above- and below-median groups of the IV versus the as-treated operative approach (Supplemental Tables 1 and 2, see http://links.lww.com/AOSO/A448). After instrument implementation, the number of covariates with standardized difference >10% was reduced compared with the as-treated covariates for robotic versus laparoscopic and robotic versus open approaches. The Durbin-Wu-Hausman test *P* value for endogeneity was <0.001 for both colon and rectal cancer. Thus, we used an IV for our primary analysis.

We compared mortality with the association of hospital volume of robotic-assisted surgeries that may reflect experience in using robotics. We calculated hospitals’ annual volume of robotic surgery and then stratified hospitals into quartiles of robotic volume to compare mortality by approach among the subgroups of hospitals. We also examined 5-year survival rate by year of cancer diagnosis that might also reflect the adoption of minimally invasive surgery during the same period that may also influence the survival trends. All analyses were performed using SAS version 9.4 (SAS Institute) and Stata version 18 (StataCorp LLC). Tests were 2-sided, and significance was set at *P* < 0.05, or 95% confidence intervals (CIs) excluding 1.

## RESULTS

During the study period, 326,406 patients underwent resection for colon cancer and 96,979 patients underwent resection for rectal cancer. Demographic data for these populations, subdivided by operative approach, are summarized for colon and rectal cancer in Tables 1 and 2, respectively. Colon and rectal cancer patients who underwent robotic-assisted resection tended to be younger at the age of diagnosis, and a larger proportion was male. Both colon and rectal patients who underwent robotic-assisted resection were more likely to have a Charlson-Deyo score of zero. Colon and rectal cancer patients who underwent emergent operative intervention were less likely to undergo a robotic-assisted approach.

**TABLE 1.** Comparison of Colon Cancer Patient Demographics Based on Operative Approach for Surgical Resection

	All	Robotic	Laparoscopic	Open	*P*
	N = 326,406	N = 23,300	N = 147,548	N = 155,558	
Age at diagnosis	69.2 (12.4)	67.2 (11.9)	68.6 (12.3)	70.0 (12.5)	<0.0001
Sex					
Male	158,767 (48.6%)	11,760 (50.5%)	71,996 (48.8%)	75,011 (48.2%)	<0.0001
Female	167,639 (51.4%)	11,540 (49.5%)	75,552 (51.2%)	80,547 (51.8%)	<0.0001
Race					
White	271,005 (83.0%)	19,224 (82.5%)	123,002 (83.4%)	128,779 (82.8%)	<0.0001
Black	38,671 (11.8%)	2557 (11.0%)	16,307 (11.1%)	19,807 (12.7%)	<0.0001
Other	16,730 (5.1%)	1519 (6.5%)	8239 (5.6%)	6972 (4.5%)	<0.0001
Hispanic	18,220 (5.6%)	1498 (6.4%)	8324 (5.6%)	8398 (5.4%)	<0.0001
Region					
Midwest	85,912 (26.3%)	5768 (24.8%)	37,106 (25.1%)	43,038 (27.7%)	<0.0001
Northeast	66,831 (20.5%)	4402 (18.9%)	32,902 (22.3%)	29,527 (19.0%)	
South	123,240 (37.8%)	9251 (39.7%)	52,358 (35.5%)	61,631 (39.6%)	
West	50,423 (15.4%)	3879 (16.6%)	25,182 (17.1%)	21,362 (13.7%)	
Urbanicity					
Metro	278,134 (85.2%)	20,567 (88.3%)	128,456 (87.1%)	129,111 (83.0%)	<0.0001
Urban	42,260 (12.9%)	2413 (10.4%)	16,653 (11.3%)	23,194 (14.9%)	
Rural	6012 (1.8%)	320 (1.4%)	2439 (1.7%)	3253 (2.1%)	
Insurance category					
Private	100,745 (30.9%)	8685 (37.3%)	49,979 (33.9%)	42,081 (27.1%)	<0.0001
Government	213,268 (65.3%)	14,052 (60.3%)	93,142 (63.1%)	106,074 (68.2%)	
Not insured	8445 (2.6%)	347 (1.5%)	2959 (2.0%)	5139 (3.3%)	
Unknown	3948 (1.2%)	216 (0.9%)	1468 (1.0%)	2264 (1.5%)	
Charlson-Deyo Score					
0	215,734 (66.1%)	16,144 (69.3%)	98,592 (66.8%)	100,998 (64.9%)	<0.0001
1	70,434 (21.6%)	4606 (19.8%)	31,222 (21.2%)	34,606 (22.2%)	
2	23,877 (7.3%)	1445 (6.2%)	10,475 (7.1%)	11,957 (7.7%)	
≥3	16,361 (5.0%)	1105 (4.7%)	7259 (4.9%)	7997 (5.1%)	
Clinical stage group					
1	111,631 (34.2%)	9012 (38.7%)	59,626 (40.4%)	42,993 (27.6%)	<0.0001
2	115,841 (35.5%)	7486 (32.1%)	47,948 (32.5%)	60,407 (38.8%)	
3	98,934 (30.3%)	6802 (29.2%)	39,974 (27.1%)	52,158 (33.5%)	
Grade					
Low grade	237,851 (72.9%)	16,450 (70.6%)	108,803 (73.7%)	112,598 (72.4%)	<0.0001
High grade	53,561 (16.4%)	2870 (12.3%)	21,901 (14.8%)	28,790 (18.5%)	
Unknown	34,994 (10.7%)	3980 (17.1%)	16,844 (11.4%)	14,170 (9.1%)	
Adenocarcinoma	324,689 (99.5%)	23,200 (99.6%)	146,880 (99.5%)	154,609 (99.4%)	<0.0001
Emergent surgery (within 3 days of diagnosis)	119,868 (36.7%)	5035 (21.6%)	47,390 (32.1%)	67,443 (43.4%)	<0.0001

**TABLE 2.** Comparison of Rectal Cancer Patient Demographics Based on Operative Approach for Surgical Resection

	All	Robotic	Laparoscopic	Open	*P*
	N = 86,979	N = 14,518	N = 33,174	N = 39,287	
Age at diagnosis	63.0 (11.7)	61.8 (11.3)	62.6 (11.8)	63.8 (11.8)	<0.0001
Male	51,533 (59.2%)	8955 (61.7%)	18,833 (56.8%)	23,745 (60.4%)	<0.0001
Female	35,446 (40.8%)	5563 (38.3%)	14,341 (43.2%)	15,542 (39.6%)	<0.0001
White	72,209 (83.0%)	12,381 (85.3%)	26,734 (80.6%)	33,094 (84.2%)	<0.0001
Black	8844 (10.2%)	1061 (7.3%)	3864 (11.6%)	3919 (10.0%)	<0.0001
Other	5926 (6.8%)	1076 (7.4%)	2576 (7.8%)	2274 (5.8%)	<0.0001
Hispanic	5893 (6.8%)	957 (6.6%)	2409 (7.3%)	2527 (6.4%)	<0.0001
Region					
Midwest	23,720 (27.3%)	4319 (29.7%)	8618 (26.0%)	10,783 (27.4%)	<0.0001
Northeast	17,007 (19.6%)	2590 (17.8%)	7496 (22.6%)	6921 (17.6%)	
South	31,918 (36.7%)	4942 (34.0%)	10,998 (33.2%)	15,978 (40.7%)	
West	14,334 (16.5%)	2667 (18.4%)	6062 (18.3%)	5605 (14.3%)	
Urbanicity					
Metro	72,257 (83.1%)	12,106 (83.4%)	28,398 (85.6%)	31,753 (80.8%)	<0.0001
Urban	13,022 (15.0%)	2152 (14.8%)	4247 (12.8%)	6623 (16.9%)	
Rural	1700 (2.0%)	260 (1.8%)	529 (1.6%)	911 (2.3%)	
Insurance category					
Private	39,930 (45.9%)	7405 (51.0%)	16,156 (48.7%)	16,369 (41.7%)	<0.0001
Government	43,018 (49.5%)	6684 (46.0%)	15,760 (47.5%)	20,574 (52.4%)	
Not insured	2852 (3.3%)	290 (2.0%)	915 (2.8%)	1647 (4.2%)	
Unknown	1179 (1.4%)	139 (1.0%)	343 (1.0%)	697 (1.8%)	
Charlson-Deyo Score					
0	65,011 (74.7%)	11,137 (76.7%)	25,170 (75.9%)	28,704 (73.1%)	<0.0001
1	15,539 (17.9%)	2434 (16.8%)	5607 (16.9%)	7498 (19.1%)	
2	4077 (4.7%)	608 (4.2%)	1478 (4.5%)	1991 (5.1%)	
≥3	2352 (2.7%)	339 (2.3%)	919 (2.8%)	1094 (2.8%)	
Clinical stage group					
1	33,615 (38.6%)	3458 (23.8%)	18,164 (54.8%)	11,993 (30.5%)	<0.0001
2	23,158 (26.6%)	4374 (30.1%)	6503 (19.6%)	12,281 (31.3%)	
3	30,206 (34.7%)	6686 (46.1%)	8507 (25.6%)	15,013 (38.2%)	
Grade					
Low grade	64,625 (74.3%)	10,700 (73.7%)	24,582 (74.1%)	29,343 (74.7%)	<0.0001
High grade	7842 (9.0%)	1256 (8.7%)	2373 (7.2%)	4213 (10.7%)	
Unknown	14,512 (16.7%)	2562 (17.6%)	6219 (18.7%)	5731 (14.6%)	
Adenocarcinoma	85,700 (98.5%)	14,390 (99.1%)	32,692 (98.5%)	38,618 (98.3%)	<0.0001
Emergent surgery (within 3 days of diagnosis)	14,116 (16.2%)	503 (3.5%)	9121 (27.5%)	4492 (11.4%)	<0.0001

**FIGURE 1.**
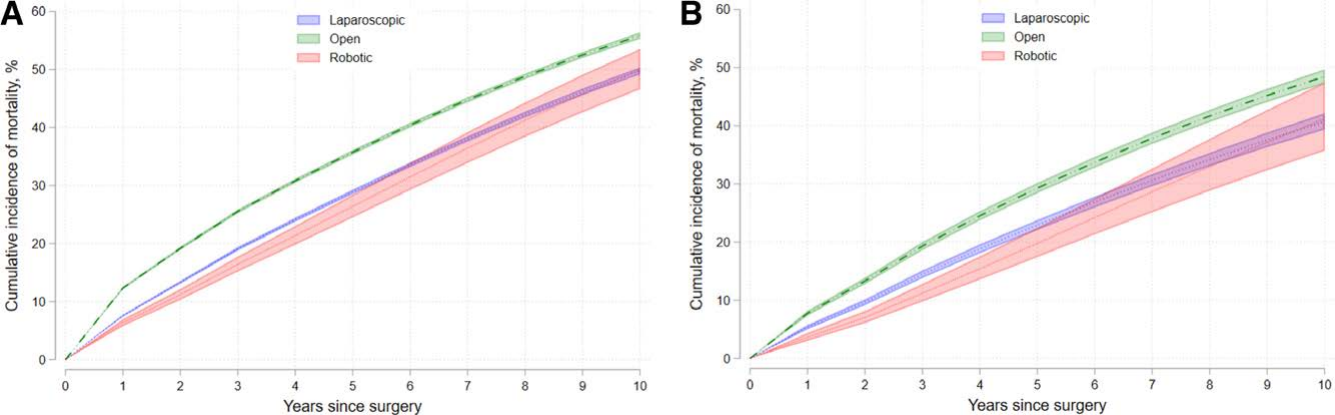
Cumulative incidence of mortality for all stages of colon (A) and rectal (B) cancer after resection.

After risk adjustment and running our IV proportional hazards model, we found that the cumulative incidence of mortality was lowest for patients undergoing robotic surgery for resection of their stages 1 to 3 colon and rectal cancer (Table 3). The 5-year cumulative incidence of mortality for colon and rectal cancer was highest for patients who underwent open approaches (35.73 [35.37–36.1] and 39.27 [28.44–30.13], respectively), compared with lower mortality for those undergoing laparoscopic (28.91 [28.55–29.27] and 22.93 [22.11–23.78], respectively) and robotic approaches (26.39 [24.51–28.42] and 19.77 [17.32–22.43], respectively). These data are summarized graphically in Figure 1.

**TABLE 3.** Cumulative Incidence of Mortality for Overall (Includes Stages 1–3) Colon and Rectal Cancer Patients Following Surgical Resection

Colon Cancer			
Time (Years)	Lap	Open	Robotic
1	7.58 (7.42–7.73)	12.33 (12.15–12.52)	6.34 (5.78–6.94)
2	13.33 (13.11–13.55)	19.17 (18.93–19.41)	11.2 (10.32–12.16)
3	19.09 (18.81–19.37)	25.54 (25.25–25.84)	16.42 (15.19–17.74)
4	24.11 (23.79–24.44)	30.82 (30.49–31.15)	21.37 (19.82–23.04)
5	28.91 (28.55–29.27)	35.73 (35.37–36.1)	26.39 (24.51–28.42)
**Rectal Cancer**			
**Time (Years**)	**Lap**	**Open**	**Robotic**
1	5.36 (5.02–5.73)	7.75 (7.36–8.17)	3.64 (3.03–4.37)
2	9.64 (9.17–10.14)	13.31 (12.79–13.86)	7.04 (6.09–8.13)
3	14.45 (13.83–15.1)	19.3 (18.63–19.98)	11.2 (9.8–12.8)
4	18.82 (18.09–19.58)	24.52 (23.76–25.31)	15.43 (13.57–17.55)
5	22.93 (22.11–23.78)	29.27 (28.44–30.13)	19.77 (17.43–22.43)

Based on our model, we also calculated adjusted mortality rate HRs for laparoscopic and open approaches compared with robotic approaches for both colon and rectal cancer resections (Table 4). Mortality rate HRs were higher in the early postoperative phase following colon and rectal resection. For example, for colon cancer, laparoscopic and open 1-year mortality HRs were 1.23 (95% CI: 1.11–1.35) and 1.78 (95% CI: 1.61–1.96), respectively, but 5-year mortality HRs were 0.98 (95% CI: 0.87–1.12) and 1.16 (1.02–1.32), respectively. Similarly for rectal cancer resections, mortality HRs at 1 year were 1.42 (95% CI: 1.16–1.73) and 2.03 (95% CI: 1.67–2.48) for laparoscopic and open resections, respectively, whereas at 5 years, these hazard radios were 1.00 (95% CI: 0.79–1.25) and 1.31 (95% CI: 1.04–1.64), respectively.

**TABLE 4.** Mortality Rate Hazard Ratios for Laparoscopic and Open Approaches for Colon and Rectal Cancer Compared With Robotic Approach

Time (Years)	Colon Cancer		Rectal Cancer	
	Lap	Open	Lap	Open
	HR (95% CI)	HR (95% CI)	HR (95% CI)	HR (95% CI)
1	1.23 (1.11–1.35)	1.78 (1.61–1.96)	1.42 (1.16–1.73)	2.03 (1.67–2.48)
2	1.2 (1.09–1.33)	1.59 (1.44–1.75)	1.32 (1.09–1.6)	1.84 (1.53–2.22)
3	1.12 (1.01–1.24)	1.38 (1.24–1.53)	1.21 (1–1.47)	1.64 (1.36–1.99)
4	1.04 (0.92–1.16)	1.24 (1.1–1.4)	1.09 (0.89–1.33)	1.45 (1.19–1.77)
5	0.98 (0.87–1.12)	1.16 (1.02–1.32)	1 (0.79–1.25)	1.31 (1.04–1.64)

**FIGURE 2.**
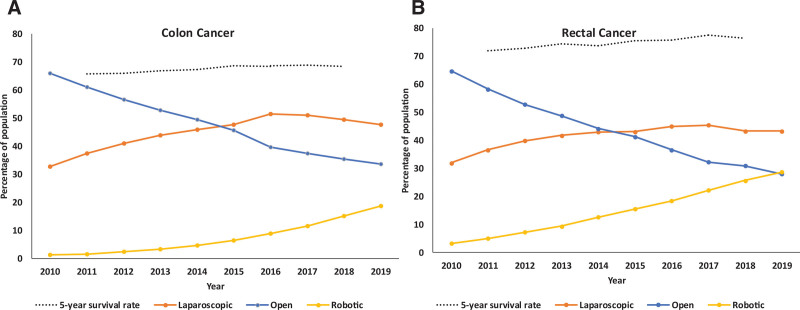
Five-year survival rates for colon (A) and rectal (B) cancer patients. Temporal changes in rates of surgical approach for colon (A) and rectal (B).

Figure 2 and Supplemental Figs. 1 and 2, see http://links.lww.com/AOSO/A448, display the trends in utilization of different surgical approaches for colon and rectal cancer during the study. Data for colon and rectal cancer and rates of change in surgical approach were plotted with changes in rates of 5-year survival and are represented in Figure 2 as well. Substantial growth in the proportion of minimally invasive cases was observed during the study period. For example, the proportion of laparoscopic and robotic cases for colon resections increased from 32.7% to 47.8% and 1.2% to 18.6%, respectively. Similar trends were observed for rectal cancer but with even greater growth in robotic-assisted surgery.

Using our risk adjustment and IV model, we also performed sensitivity analyses for stage 1 and 3 colon and rectal cancer resection patients. These findings are detailed in Supplemental Tables 3–6 and Supplemental Figures 3–6, see http://links.lww.com/AOSO/A448.

## DISCUSSION

This IV analysis of long-term survival among NCDB patients with colon or rectal cancer had 3 principal findings. First, the data here redemonstrate the significant evolution of surgical approach toward minimally invasive approaches in the operative management of colorectal cancer. Additionally, the increased uptake of minimally invasive approaches did not uncover evidence of adverse effects on overall survival among colorectal cancer patients. Finally, these findings did demonstrate that minimally invasive approaches were associated with better long-term survival rates compared with open surgical management for colon and rectal cancer. Taken together, the changes in the surgical management of colon and rectal cancer toward minimally invasive approaches (eg, laparoscopic and robotic) have not adversely affected and may demonstrate some benefits for long-term survival among colorectal cancer patients.

Existing data suggest that the shift away from open operative approaches has been driven by laparoscopy and robotics for different reasons. For example, the primary drivers of increased uptake in laparoscopy have been for abdominal colon operations (eg, partial or total colectomy, colostomy, or colostomy takedown), whereas robotic volume has been driven by pelvic operations (eg, abdominoperineal resection, low anterior resection, or proctocolectomy).^24^ Multiple studies have demonstrated that significant variation across regions and centers remains with respect to the uptake of minimally invasive approaches and the extent of their growth.^25–28^ While there is variation across studies based on study scope and database selection, it is likely that open operative approaches for colorectal cancer now comprise less than 50% of operative volume.^11,24,25,27,29^ Our data support the suspected large growth of robotics as a modality in colorectal cancer surgery, given that at the end of the study period, robotic approaches comprised nearly 20% and 30% of the case volume for colon and rectal cancer, respectively. For comparison, at the outset of our study period in 2010, robotic approaches comprised less than 5% of the operative volume for both colon and rectal cancer operations in our data.

This study has important implications for minimally invasive surgical approaches and its efficacy in cancer surgery. In 2018, Melamed et al^12^ used inverse probability weighting in a large cohort study of patients with early-stage cervical cancer and found that minimally invasive radical hysterectomy was associated with shorter overall survival than open surgery. Early randomized trials demonstrated the safety of video-assisted thoracic surgery compared with thoracotomy for lung cancer demonstrated its safety and efficacy. However, data are more sparse comparing newer robotic platform outcomes, although early data suggest its equivalence.^30–33^ Minimally invasive gastrectomy for gastric cancer was found to portend better long-term survival than open approaches.^34^ The findings in our study did not demonstrate any evidence of minimally invasive approaches untoward effects on overall survival for the surgical management of colon or rectal cancer. These findings underscore that there is no appreciable long-term downside to favoring continued expansion of minimally invasive approaches, when clinically feasible, from a long-term survival standpoint. In the future, other abdominal cancers could be considered for similar study where the surgical approach is highly contested, although findings may be more limited due to case volume such as in the case of pancreatic cancer and minimally invasive versus open pancreaticoduodenectomy, although recent propensity score matching data favored improved overall survival and disease-free survival for minimally invasive pancreas surgery compared with open surgery among pancreatic cancer patients.^35^

The improved long-term survival for both laparoscopic and robotic-assisted colon and rectal cancer surgery raises the question of whether surgical approach affects the survival of these patients. On the one hand, there have been improvements in overall survival, which coincides with the timeline of the paradigm shift from open to minimally invasive approaches for colon and rectal cancer as evidenced by Figure 2. However, if operative approach was a primary factor in driving rates of long-term survival, we would expect to see a clearer relationship between survival and uptake of minimally invasive practices in Figure 2. We suspect that improving overall survival is a product of neoadjuvant and adjuvant therapies and broader access to preventive and screening services, as well as other patient variables that, despite our approach, were unable to be accounted for.

These findings should be interpreted in the context of important limitations in this study. First, an important issue in minimally invasive approaches for surgical management of oncologic disease is concern for local recurrence, which is notoriously challenging to track in large databases such as the NCDB used in our study. While local recurrence was unable to be measured in this study, if they are occurring, they do not appear to be overtly impactful on important long-term outcome variables (eg, long-term survival). In addition, selection bias (eg, using the NCDB as our data source, which is not a population-based database) is an important limitation in all observational studies. However, this study was designed with an IV analysis to address this specific important limitation to provide real-world, bias-free assessments of outcomes in clinical practice. Further, we confirmed the strength and validity of our instrument were appropriate. Specific to using the NCDB and associated potential selection bias based on geographic regions, we also excluded patients in referral regions where no robotic-assisted colon or rectal cancer operations occurred during the study period. Finally, this study was limited by its study period including the years 2011 to 2018, which is limited to demonstrate the contemporary growth of robotic surgery in most recent years. However, this study included the most recent data available with appropriate follow-up and power to identify differences in survival based on surgical approach.

## CONCLUSION

In this study, we used an IV analysis to minimize selection bias while evaluating broad, national, real-world practice in colon and rectal cancer surgery. Patients who underwent minimally invasive surgical approaches had improved long-term survival, compared with their open surgery counterparts. However, long-term survival changes did not correlate with the large expansion of minimally invasive approaches, which suggests that growing these approaches is not a viable strategy to improve long-term patient outcomes.

## Supplementary Material


