# Supplementary material for: Surgical Approach and Variation in Long-Term Survival Following Colorectal Cancer Surgery Using Instrumental Variable Analysis
Source: Ann Surg Open. 2025 Jan 23;6(1):e538. doi: 10.1097/AS9.0000000000000538 (PMC11932609; doi:10.1097/AS9.0000000000000538)
Supplement: Supplementary file 1 [file as9-6-e538-s001.pdf]

Supplemental Information

	Actual treatment							Prior year Robotic utilization rate			
	Robotic	Open	Laparoscopic	Standardized difference (%)				Above median	Below median	Standardized difference	P-value
				Robotic vs. Open	P-value	Robotic vs. Laparoscopic	P-value				
N	N=23,300	N=155,558	N=147,548					N=160,593	N=165,813		
Age at Diagnosis	67.2 (11.9)	70.0 (12.5)	68.6 (12.3)	23.5	<0.0001	11.5	<0.0001	68.9 (12.4)	69.4 (12.4)	3.7	<0.0001
Female	11,540 (49.5%)	80,547 (51.8%)	75,552 (51.2%)	4.5	<0.0001	3.4	<0.0001	82,086 (51.1%)	85,553 (51.6%)	1.0	0.006
Race											
White	19,224 (82.5%)	128,779 (82.8%)	123,002 (83.4%)	0.7	0.294	2.3	0.001	130,967 (81.6%)	140,038 (84.5%)	7.7	<0.0001
Black	2,557 (11.0%)	19,807 (12.7%)	16,307 (11.1%)	5.4	<0.0001	0.2	0.725	19,911 (12.4%)	18,760 (11.3%)	3.4	<0.0001
Other	1,519 (6.5%)	6,972 (4.5%)	8,239 (5.6%)	8.9	<0.0001	3.9	<0.0001	9,715 (6.0%)	7,015 (4.2%)	8.2	<0.0001
Hispanic	1,498 (6.4%)	8,398 (5.4%)	8,324 (5.6%)	4.4	<0.0001	3.3	<0.0001	10,166 (6.3%)	8,054 (4.9%)	6.4	<0.0001
Region											
Mid-west	5,768 (24.8%)	43,038 (27.7%)	37,106 (25.1%)	6.6	<0.0001	0.9	0.198	41,625 (25.9%)	44,287 (26.7%)	1.8	<0.0001
North-East	4,402 (18.9%)	29,527 (19.0%)	32,902 (22.3%)	0.2	0.748	8.4	<0.0001	33,113 (20.6%)	33,718 (20.3%)	0.7	0.044
South	9,251 (39.7%)	61,631 (39.6%)	52,358 (35.5%)	0.2	0.806	8.7	<0.0001	59,277 (36.9%)	63,963 (38.6%)	3.4	<0.0001
West	3,879 (16.6%)	21,362 (13.7%)	25,182 (17.1%)	8.1	<0.0001	1.1	0.114	26,578 (16.5%)	23,845 (14.4%)	6.0	<0.0001
1.Metro, 2.Urban, 3.Rural, 4.NA											
Metro	20,567 (88.3%)	129,111 (83.0%)	128,456 (87.1%)	15.1	<0.0001	3.7	<0.0001	142,086 (88.5%)	136,048 (82.0%)	18.2	<0.0001
Urban	2,413 (10.4%)	23,194 (14.9%)	16,653 (11.3%)	13.7	<0.0001	3.0	<0.0001	16,540 (10.3%)	25,720 (15.5%)	15.6	<0.0001
Rural	320 (1.4%)	3,253 (2.1%)	2,439 (1.7%)	5.5	<0.0001	2.3	0.002	1,967 (1.2%)	4,045 (2.4%)	9.1	<0.0001
Insurance category											
Private	8,685 (37.3%)	42,081 (27.1%)	49,979 (33.9%)	22.0	<0.0001	7.1	<0.0001	50,502 (31.4%)	50,243 (30.3%)	2.5	<0.0001
Government	14,052 (60.3%)	106,074 (68.2%)	93,142 (63.1%)	16.5	<0.0001	5.8	<0.0001	104,995 (65.4%)	108,273 (65.3%)	0.2	0.626
Not insured	347 (1.5%)	5,139 (3.3%)	2,959 (2.0%)	11.9	<0.0001	3.9	<0.0001	3,490 (2.2%)	4,955 (3.0%)	5.1	<0.0001
Unknown	216 (0.9%)	2,264 (1.5%)	1,468 (1.0%)	4.9	<0.0001	0.7	0.33	1,606 (1.0%)	2,342 (1.4%)	3.8	<0.0001
Charlson-Deyo Score											
0	16,144 (69.3%)	100,998 (64.9%)	98,592 (66.8%)	9.3	<0.0001	5.3	<0.0001	107,187 (66.7%)	108,547 (65.5%)	2.7	<0.0001
1	4,606 (19.8%)	34,606 (22.2%)	31,222 (21.2%)	6.1	<0.0001	3.5	<0.0001	31,055 (19.3%)	39,379 (23.7%)	10.7	<0.0001
2	1,445 (6.2%)	11,957 (7.7%)	10,475 (7.1%)	5.8	<0.0001	3.6	<0.0001	11,810 (7.4%)	12,067 (7.3%)	0.3	0.401
3 or more	1,105 (4.7%)	7,997 (5.1%)	7,259 (4.9%)	1.8	0.01	0.8	0.244	10,541 (6.6%)	5,820 (3.5%)	14.0	<0.0001
Clinical stage											
Stage 1	9,012 (38.7%)	42,993 (27.6%)	59,626 (40.4%)	23.6	<0.0001	3.5	<0.0001	53,242 (33.2%)	58,389 (35.2%)	4.3	<0.0001
Stage 2	7,486 (32.1%)	60,407 (38.8%)	47,948 (32.5%)	14.0	<0.0001	0.8	0.265	57,040 (35.5%)	58,801 (35.5%)	0.1	0.738
Stage 3	6,802 (29.2%)	52,158 (33.5%)	39,974 (27.1%)	9.4	<0.0001	4.7	<0.0001	50,311 (31.3%)	48,623 (29.3%)	4.4	<0.0001
Grade							<0.0001				
Low grade	16,450 (70.6%)	112,598 (72.4%)	108,803 (73.7%)	3.9	<0.0001	7.0	<0.0001	110,224 (68.6%)	127,627 (77.0%)	18.8	<0.0001
High grade	2,870 (12.3%)	28,790 (18.5%)	21,901 (14.8%)	17.2	<0.0001	7.4	<0.0001	23,518 (14.6%)	30,043 (18.1%)	9.4	<0.0001
Unknown	3,980 (17.1%)	14,170 (9.1%)	16,844 (11.4%)	23.8	<0.0001	16.3	<0.0001	26,851 (16.7%)	8,143 (4.9%)	38.7	<0.0001
Adenocarcinoma	23,200 (99.6%)	154,609 (99.4%)	146,880 (99.5%)	2.5	0.001	0.4	0.618	159,737 (99.5%)	164,952 (99.5%)	0.2	0.587
Emergent surgery (within 3 days of diagnosis)	5,035 (21.6%)	67,443 (43.4%)	47,390 (32.1%)	47.7	<0.0001	23.9	<0.0001	57,178 (35.6%)	62,690 (37.8%)	4.6	<0.0001
Median Income Quartiles											

< $40,227	3,079 (13.2%)	28,658 (18.4%)	20,841 (14.1%)	13.8	<0.0001	1.9	0.013	23,621 (14.7%)	28,957 (17.5%)	6.4	<0.0001
$40,227 - $50,353	4,031 (17.3%)	32,674 (21.0%)	26,334 (17.8%)	8.2	<0.0001	0.4	0.58	29,351 (18.3%)	33,688 (20.3%)	3.7	<0.0001
$50,354 - $63,332	4,773 (20.5%)	32,297 (20.8%)	29,886 (20.3%)	1.5	0.052	1.9	0.012	32,399 (20.2%)	34,557 (20.8%)	0.3	0.481
>=$63,333	7,784 (33.4%)	43,001 (27.6%)	50,353 (34.1%)	17.0	<0.0001	0.1	0.873	51,575 (32.1%)	49,563 (29.9%)	8.1	<0.0001
Not available	3,633 (15.6%)	18,928 (12.2%)	20,134 (13.6%)	9.9	<0.0001	5.5	<0.0001	23,647 (14.7%)	19,048 (11.5%)	9.6	<0.0001
Percent no high school degree Quartiles											
>=21.0%	3,156 (13.5%)	26,252 (16.9%)	20,785 (14.1%)	8.3	<0.0001	0.7	0.389	23,300 (14.5%)	26,893 (16.2%)	3.3	<0.0001
13.0% - 20.9%	4,788 (20.5%)	38,075 (24.5%)	31,534 (21.4%)	8.0	<0.0001	1.0	0.192	35,677 (22.2%)	38,720 (23.4%)	0.6	0.076
7.0%-12.9%	6,602 (28.3%)	44,819 (28.8%)	42,459 (28.8%)	1.6	0.059	0.5	0.618	45,756 (28.5%)	48,124 (29.0%)	1.4	<0.0001
< 7.0%	5,342 (22.9%)	28,904 (18.6%)	33,974 (23.0%)	13.9	<0.0001	1.1	0.145	33,526 (20.9%)	34,694 (20.9%)	2.1	<0.0001
Not available	3,412 (14.6%)	17,508 (11.3%)	18,796 (12.7%)	10.1	<0.0001	5.5	<0.0001	22,334 (13.9%)	17,382 (10.5%)	10.5	<0.0001

Supplemental Table 1: Instrumental variable balancing for colon cancer.

Supplemental Information

	Actual treatment							Prior year Robotic utilization rate			
	**Robotic**	**Open**	**Laparoscopic**	**Standardized difference (%)**				**Above median**	**Below median**	**Standardized difference**	**P-value**
				**Robotic vs. Open**	**P-value**	**Robotic vs. Laparoscopic**	**P-value**				
N	N=14,518	N=39,287	N=33,174					N=43,385	N=43,594		
Age at Diagnosis	61.8 (11.3)	63.8 (11.8)	62.6 (11.8)	17.4	<0.0001	7.3	<0.0001	62.7 (11.6)	63.3 (11.9)	4.9	<0.0001
Female	5,563 (38.3%)	15,542 (39.6%)	14,341 (43.2%)	2.5	0.009	10.0	<0.0001	17,591 (40.5%)	17,855 (41.0%)	0.8	0.217
Race											
White	12,381 (85.3%)	33,094 (84.2%)	26,734 (80.6%)	2.9	0.003	12.5	<0.0001	35,532 (81.9%)	36,677 (84.1%)	6.0	<0.0001
Black	1,061 (7.3%)	3,919 (10.0%)	3,864 (11.6%)	9.5	<0.0001	14.9	<0.0001	4,438 (10.2%)	4,406 (10.1%)	0.4	0.55
Other	1,076 (7.4%)	2,274 (5.8%)	2,576 (7.8%)	6.5	<0.0001	1.3	0.181	3,415 (7.9%)	2,511 (5.8%)	8.4	<0.0001
Hispanic	957 (6.6%)	2,527 (6.4%)	2,409 (7.3%)	0.6	0.504	2.6	0.009	3,261 (7.5%)	2,632 (6.0%)	5.9	<0.0001
Region											
Mid-west	4,319 (29.7%)	10,783 (27.4%)	8,618 (26.0%)	5.1	<0.0001	8.4	<0.0001	12,637 (29.1%)	11,083 (25.4%)	8.3	<0.0001
North-East	2,590 (17.8%)	6,921 (17.6%)	7,496 (22.6%)	0.6	0.547	11.9	<0.0001	8,289 (19.1%)	8,718 (20.0%)	2.3	0.001
South	4,942 (34.0%)	15,978 (40.7%)	10,998 (33.2%)	13.7	<0.0001	1.9	0.059	14,269 (32.9%)	17,649 (40.5%)	15.8	<0.0001
West	2,667 (18.4%)	5,605 (14.3%)	6,062 (18.3%)	11.1	<0.0001	0.3	0.801	8,190 (18.9%)	6,144 (14.1%)	12.9	<0.0001
1.Metro, 2.Urban, 3.Rural, 4.NA											
Metro	12,106 (83.4%)	31,753 (80.8%)	28,398 (85.6%)	6.7	<0.0001	6.1	<0.0001	36,277 (83.6%)	35,980 (82.5%)	2.9	<0.0001
Urban	2,152 (14.8%)	6,623 (16.9%)	4,247 (12.8%)	5.6	<0.0001	5.9	<0.0001	6,316 (14.6%)	6,706 (15.4%)	2.3	0.001
Rural	260 (1.8%)	911 (2.3%)	529 (1.6%)	3.7	<0.0001	1.5	0.122	792 (1.8%)	908 (2.1%)	1.9	0.006
Insurance category											
Private	7,405 (51.0%)	16,369 (41.7%)	16,156 (48.7%)	18.8	<0.0001	4.6	<0.0001	20,443 (47.1%)	19,487 (44.7%)	4.9	<0.0001
Government	6,684 (46.0%)	20,574 (52.4%)	15,760 (47.5%)	12.7	<0.0001	2.9	0.003	21,428 (49.4%)	21,590 (49.5%)	0.3	0.691
Not insured	290 (2.0%)	1,647 (4.2%)	915 (2.8%)	12.7	<0.0001	5.0	<0.0001	1,092 (2.5%)	1,760 (4.0%)	8.5	<0.0001
Unknown	139 (1.0%)	697 (1.8%)	343 (1.0%)	7.0	<0.0001	0.8	0.442	422 (1.0%)	757 (1.7%)	6.6	<0.0001
Charlson-Deyo Score											
0	11,137 (76.7%)	28,704 (73.1%)	25,170 (75.9%)	8.4	<0.0001	2.0	0.048	32,635 (75.2%)	32,376 (74.3%)	2.2	0.001
1	2,434 (16.8%)	7,498 (19.1%)	5,607 (16.9%)	6.1	<0.0001	0.4	0.714	7,189 (16.6%)	8,350 (19.2%)	6.7	<0.0001
2	608 (4.2%)	1,991 (5.1%)	1,478 (4.5%)	4.2	<0.0001	1.3	0.189	2,040 (4.7%)	2,037 (4.7%)	0.1	0.837
3 or more	339 (2.3%)	1,094 (2.8%)	919 (2.8%)	2.8	0.004	2.8	0.006	1,521 (3.5%)	831 (1.9%)	9.9	<0.0001
Clinical stage											
Stage 1	3,458 (23.8%)	11,993 (30.5%)	18,164 (54.8%)	15.1	<0.0001	66.8	<0.0001	16,250 (37.5%)	17,365 (39.8%)	4.9	<0.0001
Stage 2	4,374 (30.1%)	12,281 (31.3%)	6,503 (19.6%)	2.5	0.012	24.5	<0.0001	10,900 (25.1%)	12,258 (28.1%)	6.8	<0.0001
Stage 3	6,686 (46.1%)	15,013 (38.2%)	8,507 (25.6%)	15.9	<0.0001	43.6	<0.0001	16,235 (37.4%)	13,971 (32.0%)	11.3	<0.0001
Grade											
Low grade	10,700 (73.7%)	29,343 (74.7%)	24,582 (74.1%)	2.3	0.02	0.9	0.361	31,855 (73.4%)	32,770 (75.2%)	4.0	<0.0001
High grade	1,256 (8.7%)	4,213 (10.7%)	2,373 (7.2%)	7.0	<0.0001	5.6	<0.0001	3,514 (8.1%)	4,328 (9.9%)	6.4	<0.0001
Unknown	2,562 (17.6%)	5,731 (14.6%)	6,219 (18.7%)	8.3	<0.0001	2.9	0.004	8,016 (18.5%)	6,496 (14.9%)	9.6	<0.0001
Adenocarcinoma	14,390 (99.1%)	38,618 (98.3%)	32,692 (98.5%)	7.3	<0.0001	5.3	<0.0001	42,766 (98.6%)	42,934 (98.5%)	0.7	0.285
Emergent surgery (within 3 days of diagnosis)	503 (3.5%)	4,492 (11.4%)	9,121 (27.5%)	30.7	<0.0001	70.4	<0.0001	6,878 (15.9%)	7,238 (16.6%)	2.0	0.003

Supplemental Information

Median Income Quartiles											
< $40,227	1,980 (13.6%)	7,054 (18.0%)	4,873 (14.7%)	11.6	<0.0001	2.3	0.035	7,404 (17.0%)	6,503 (15.0%)	4.5	<0.0001
$40,227 - $50,353	2,613 (18.0%)	8,162 (20.8%)	6,114 (18.4%)	6.1	<0.0001	0.0	0.973	8,845 (20.3%)	8,044 (18.5%)	3.2	<0.0001
$50,354 - $63,332	2,904 (20.0%)	8,021 (20.4%)	6,604 (19.9%)	0.6	0.577	1.5	0.154	8,996 (20.6%)	8,533 (19.7%)	1.0	0.172
>=$63,333	4,741 (32.7%)	10,924 (27.8%)	11,024 (33.2%)	14.2	<0.0001	0.4	0.683	12,914 (29.6%)	13,775 (31.8%)	7.4	<0.0001
Not available	2,280 (15.7%)	5,126 (13.0%)	4,559 (13.7%)	7.6	<0.0001	5.5	<0.0001	5,435 (12.5%)	6,530 (15.1%)	7.5	<0.0001
Percent no high school degree Quartiles											
>=21.0%	1,980 (13.6%)	6,810 (17.3%)	5,078 (15.3%)	9.7	<0.0001	4.2	<0.0001	6,649 (15.3%)	7,219 (16.6%)	2.1	0.004
13.0% - 20.9%	3,063 (21.1%)	9,416 (24.0%)	7,120 (21.5%)	5.8	<0.0001	0.2	0.973	9,512 (21.9%)	10,087 (23.1%)	1.3	0.074
7.0%-12.9%	4,052 (27.9%)	10,905 (27.8%)	9,450 (28.5%)	2.5	0.029	0.0	0.918	11,986 (27.6%)	12,421 (28.5%)	0.1	0.909
< 7.0%	3,297 (22.7%)	7,432 (18.9%)	7,266 (21.9%)	12.0	<0.0001	3.4	0.001	9,089 (20.9%)	8,906 (20.4%)	3.2	<0.0001
Not available	2,126 (14.6%)	4,724 (12.0%)	4,260 (12.8%)	7.7	<0.0001	5.2	<0.0001	6,149 (14.2%)	4,961 (11.4%)	8.4	<0.0001

Supplemental Table 2: Instrumental variable balancing for rectal cancer.

Supplemental Information

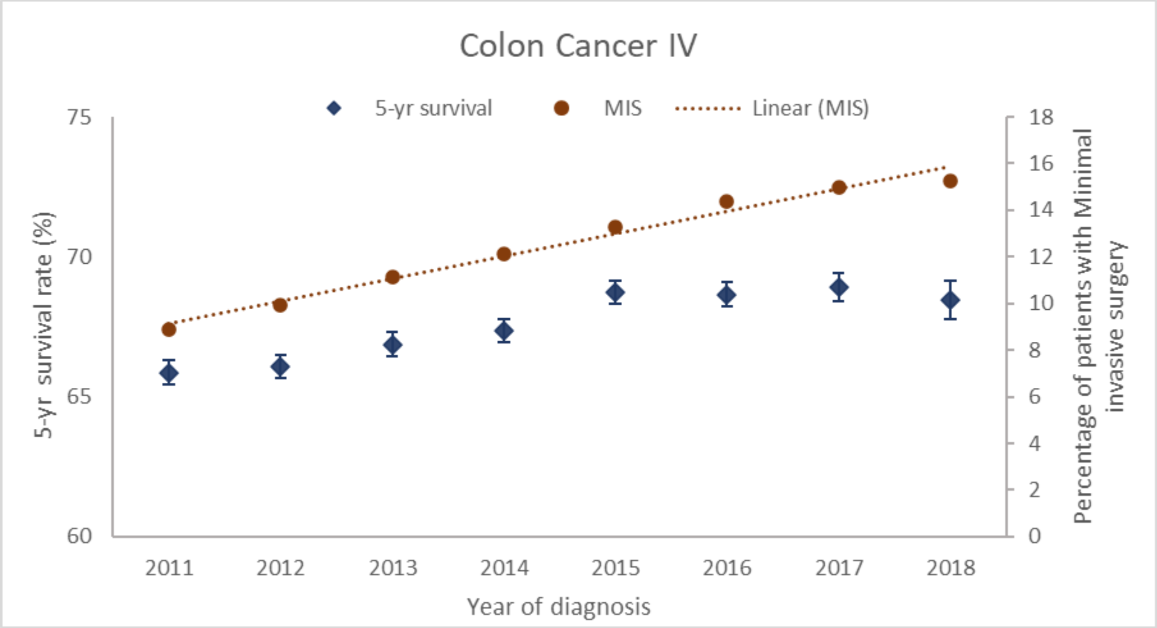

Supplemental Figure 1: Adjusted 5-year colon cancer survival for and percentage of patients who underwent minimally invasive surgery for colon cancer resection based on year of diagnosis.

Supplemental Information

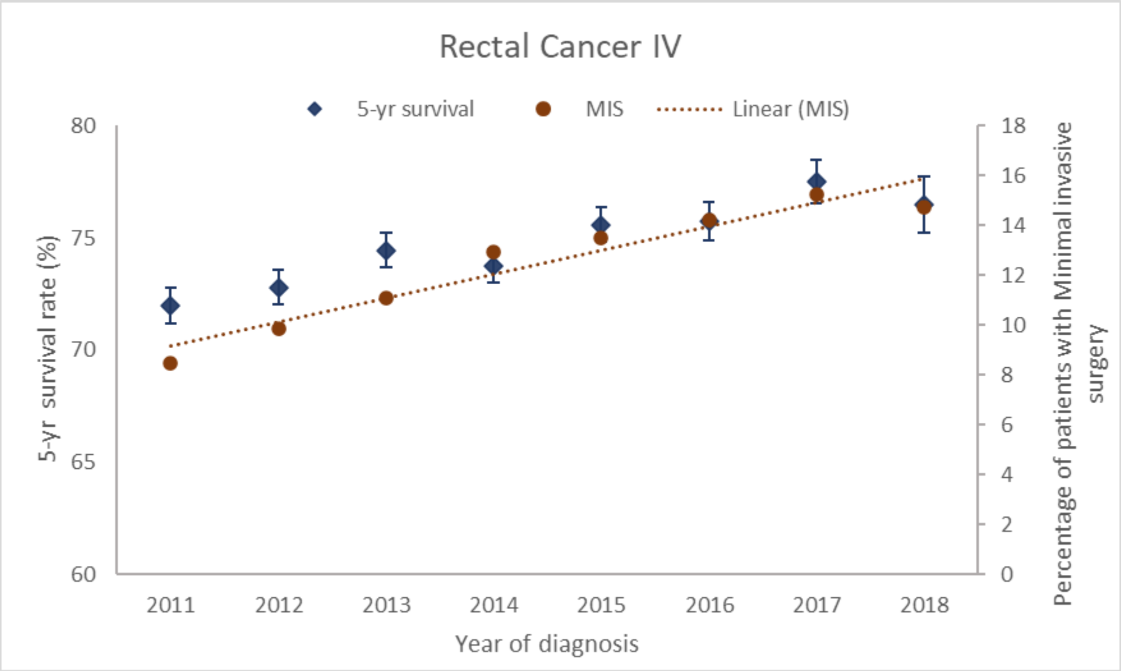

Supplemental Figure 2: Adjusted 5-year rectal cancer survival for and percentage of patients who underwent minimally invasive surgery for rectal cancer resection based on year of diagnosis.

Supplemental Information

	Colon Cancer			Rectal Cancer		
Time (Years)	Lap	Open	Robotic	Lap	Open	Robotic
1	4.7 (4.54-4.88)	7.85 (7.61-8.1)	4.35 (3.69-5.13)	3 (2.74-3.29)	4.8 (4.45-5.19)	2.9 (2.19-3.85)
2	7.81 (7.59-8.03)	11.53 (11.24-11.83)	7.09 (6.12-8.23)	5.46 (5.1-5.84)	7.81 (7.35-8.3)	4.87 (3.82-6.2)
3	11.79 (11.51-12.07)	16.09 (15.73-16.45)	10.76 (9.33-12.4)	8.55 (8.09-9.04)	11.34 (10.77-11.94)	7.34 (5.85-9.21)
4	15.75 (15.42-16.08)	20.4 (19.99-20.82)	14.68 (12.76-16.88)	12.01 (11.43-12.62)	15.11 (14.42-15.83)	10.3 (8.28-12.8)
5	19.71 (19.32-20.1)	24.62 (24.15-25.09)	18.9 (16.43-21.74)	15.48 (14.79-16.19)	18.76 (17.97-19.59)	13.54 (10.96-16.73)
6	23.93 (23.48-24.38)	29.04 (28.52-29.57)	23.59 (20.48-27.18)	18.89 (18.09-19.72)	22.27 (21.37-23.2)	16.98 (13.77-20.94)
7	28.29 (27.77-28.82)	33.53 (32.95-34.13)	28.59 (24.78-32.97)	22.21 (21.28-23.17)	25.62 (24.6-26.67)	20.53 (16.62-25.35)
8	32.63 (32-33.27)	37.92 (37.24-38.61)	33.65 (29.16-38.84)	25.42 (24.31-26.57)	28.8 (27.62-30.02)	24.09 (19.42-29.88)
9	36.84 (36.08-37.63)	42.09 (41.29-42.9)	38.6 (33.46-44.53)	28.49 (27.14-29.91)	31.8 (30.4-33.26)	27.61 (22.14-34.42)
10	40.84 (39.93-41.78)	45.97 (45.05-46.92)	43.32 (37.61-49.9)	31.44 (29.81-33.15)	34.64 (32.99-36.37)	31.03 (24.77-38.88)

Supplemental Table 3: Cumulative incidence of mortality for stage 1 colon and rectal cancer patients following surgical resection.

Supplemental Information

	Colon Cancer			Rectal Cancer		
Time (Years)	Lap	Open	Robotic	Lap	Open	Robotic
1	7.94 (7.58-8.31)	12.68 (12.27-13.1)	6.96 (5.55-8.74)	6.23 (5.28-7.35)	7.45 (6.59-8.42)	5.42 (3.77-7.8)
2	14.31 (13.84-14.8)	20.01 (19.51-20.52)	12.77 (10.44-15.62)	11.44 (10.09-12.96)	13.22 (11.98-14.58)	10.29 (7.46-14.19)
3	20.72 (20.13-21.32)	26.9 (26.31-27.49)	19.03 (15.73-23.02)	17.7 (15.86-19.76)	20.3 (18.63-22.12)	16.62 (12.27-22.5)
4	26.22 (25.58-26.89)	32.57 (31.93-33.22)	24.87 (20.69-29.88)	22.32 (20.18-24.68)	26.09 (24.14-28.19)	22.08 (16.47-29.6)
5	31.23 (30.52-31.95)	37.62 (36.93-38.31)	30.46 (25.45-36.45)	26.21 (23.85-28.8)	31.51 (29.33-33.85)	27.31 (20.5-36.39)
6	35.83 (35.06-36.62)	42.18 (41.46-42.91)	35.78 (29.98-42.71)	29.86 (27.28-32.68)	36.86 (34.5-39.4)	32.57 (24.55-43.22)
7	40.06 (39.19-40.94)	46.29 (45.51-47.08)	40.77 (34.22-48.58)	33.29 (30.41-36.44)	42.02 (39.45-44.76)	37.72 (28.5-49.93)
8	43.93 (42.94-44.95)	50.01 (49.15-50.89)	45.4 (38.18-53.99)	36.5 (33.23-40.1)	46.89 (44.05-49.92)	42.65 (32.27-56.37)
9	47.49 (46.35-48.66)	53.37 (52.41-54.36)	49.67 (41.86-58.93)	39.51 (35.76-43.65)	51.42 (48.25-54.81)	47.31 (35.86-62.42)
10	50.75 (49.46-52.08)	56.42 (55.34-57.52)	53.58 (45.28-63.41)	42.33 (38.07-47.06)	55.6 (52.06-59.38)	51.66 (39.25-67.99)

Supplemental Table 4: Cumulative incidence of mortality for stage 3 colon and rectal cancer patients following surgical resection.

	Colon Cancer		Rectal Cancer	
Time (Years)	Lap	Open	Lap	Open
	HR (95% CI)	HR (95% CI)	HR (95% CI)	HR (95% CI)
1	1.13 (0.95-1.33)	1.57 (1.32-1.86)	1.21 (0.91-1.6)	1.65 (1.24-2.18)
2	1.13 (0.95-1.35)	1.47 (1.23-1.75)	1.29 (0.96-1.73)	1.6 (1.19-2.14)
3	1.07 (0.89-1.29)	1.31 (1.09-1.58)	1.25 (0.94-1.67)	1.5 (1.12-2)
4	0.98 (0.79-1.21)	1.14 (0.91-1.41)	1.17 (0.87-1.57)	1.36 (1.01-1.82)
5	0.9 (0.7-1.16)	1.03 (0.8-1.34)	1.08 (0.78-1.49)	1.23 (0.89-1.7)
6	0.85 (0.64-1.13)	0.98 (0.73-1.3)	1.01 (0.7-1.46)	1.13 (0.79-1.63)
7	0.82 (0.6-1.12)	0.93 (0.68-1.27)	0.96 (0.64-1.45)	1.06 (0.71-1.6)
8	0.79 (0.56-1.1)	0.89 (0.63-1.25)	0.93 (0.59-1.45)	1.01 (0.64-1.57)
9	0.76 (0.53-1.09)	0.85 (0.59-1.22)	0.89 (0.55-1.45)	0.96 (0.59-1.55)
10	0.74 (0.51-1.08)	0.82 (0.56-1.2)	0.87 (0.52-1.46)	0.92 (0.55-1.54)

Supplemental Table 5: Mortality rate hazard ratios for laparoscopic and open approaches for colon and rectal cancer compared to robotic approach among patients with stage 1 disease.

Supplemental Information

	Colon Cancer		Rectal Cancer	
Time (Years)	Lap	Open	Lap	Open
	HR (95% CI)	HR (95% CI)	HR (95% CI)	HR (95% CI)
1	1.15 (0.9-1.46)	1.56 (1.22-1.98)	1.14 (0.73-1.77)	1.35 (0.88-2.08)
2	1.1 (0.86-1.41)	1.39 (1.09-1.78)	1.09 (0.71-1.68)	1.26 (0.83-1.92)
3	1.02 (0.79-1.32)	1.23 (0.95-1.6)	1 (0.64-1.54)	1.2 (0.78-1.84)
4	0.95 (0.72-1.27)	1.13 (0.85-1.5)	0.87 (0.55-1.36)	1.16 (0.74-1.81)
5	0.9 (0.66-1.24)	1.06 (0.77-1.45)	0.77 (0.47-1.26)	1.14 (0.7-1.84)
6	0.86 (0.61-1.22)	1 (0.71-1.42)	0.7 (0.4-1.2)	1.12 (0.66-1.89)
7	0.83 (0.57-1.21)	0.96 (0.66-1.39)	0.65 (0.36-1.17)	1.11 (0.63-1.95)
8	0.81 (0.54-1.21)	0.92 (0.62-1.37)	0.6 (0.32-1.15)	1.09 (0.6-2)
9	0.79 (0.52-1.2)	0.89 (0.59-1.35)	0.57 (0.29-1.13)	1.08 (0.57-2.05)
10	0.77 (0.5-1.2)	0.86 (0.56-1.34)	0.55 (0.27-1.12)	1.08 (0.55-2.09)

Supplemental Table 6: Mortality rate hazard ratios for laparoscopic and open approaches for colon and rectal cancer compared to robotic approach among patients with stage 3 disease.

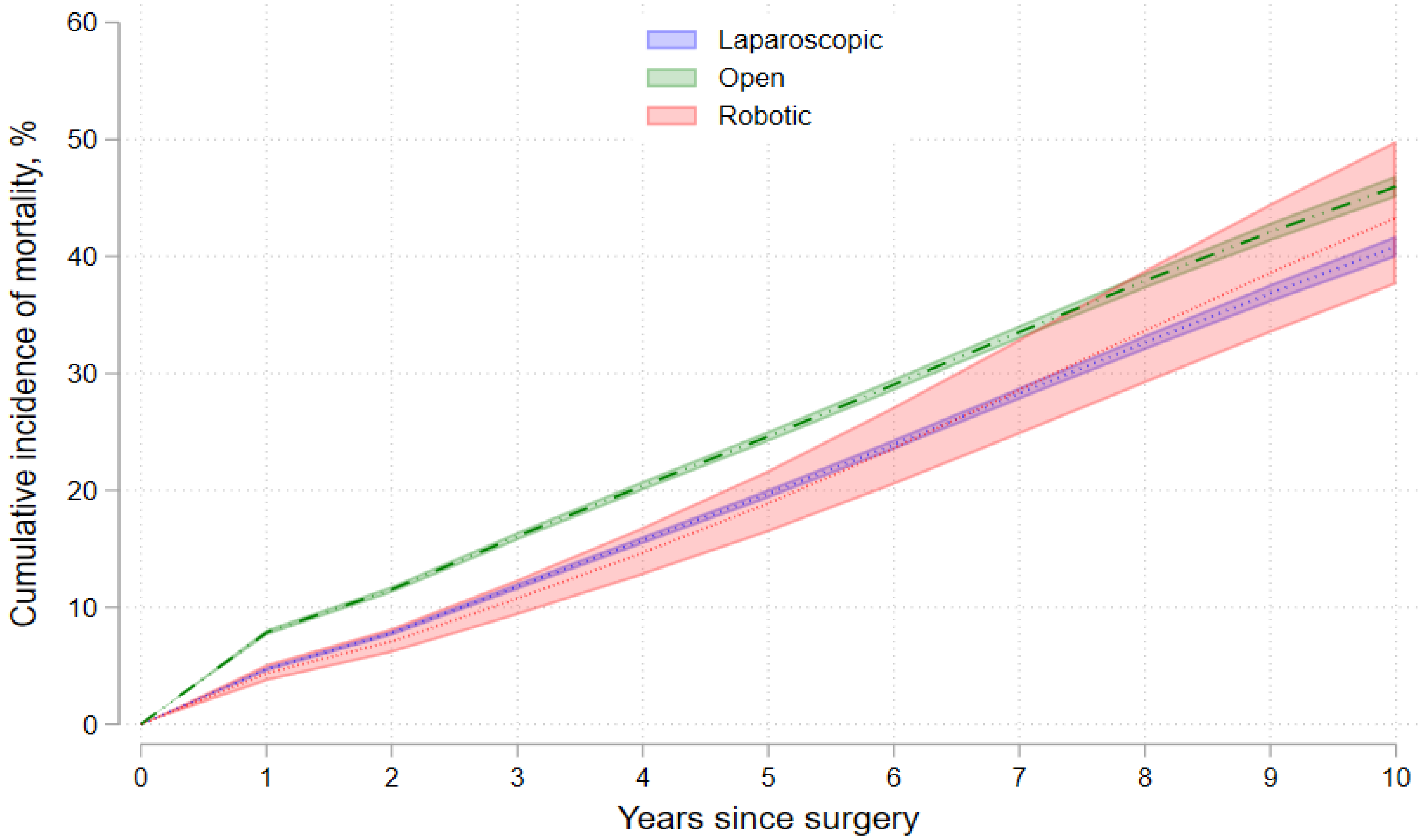

Supplemental Figure 3: Cumulative incidence of mortality for stage 1 colon cancer.

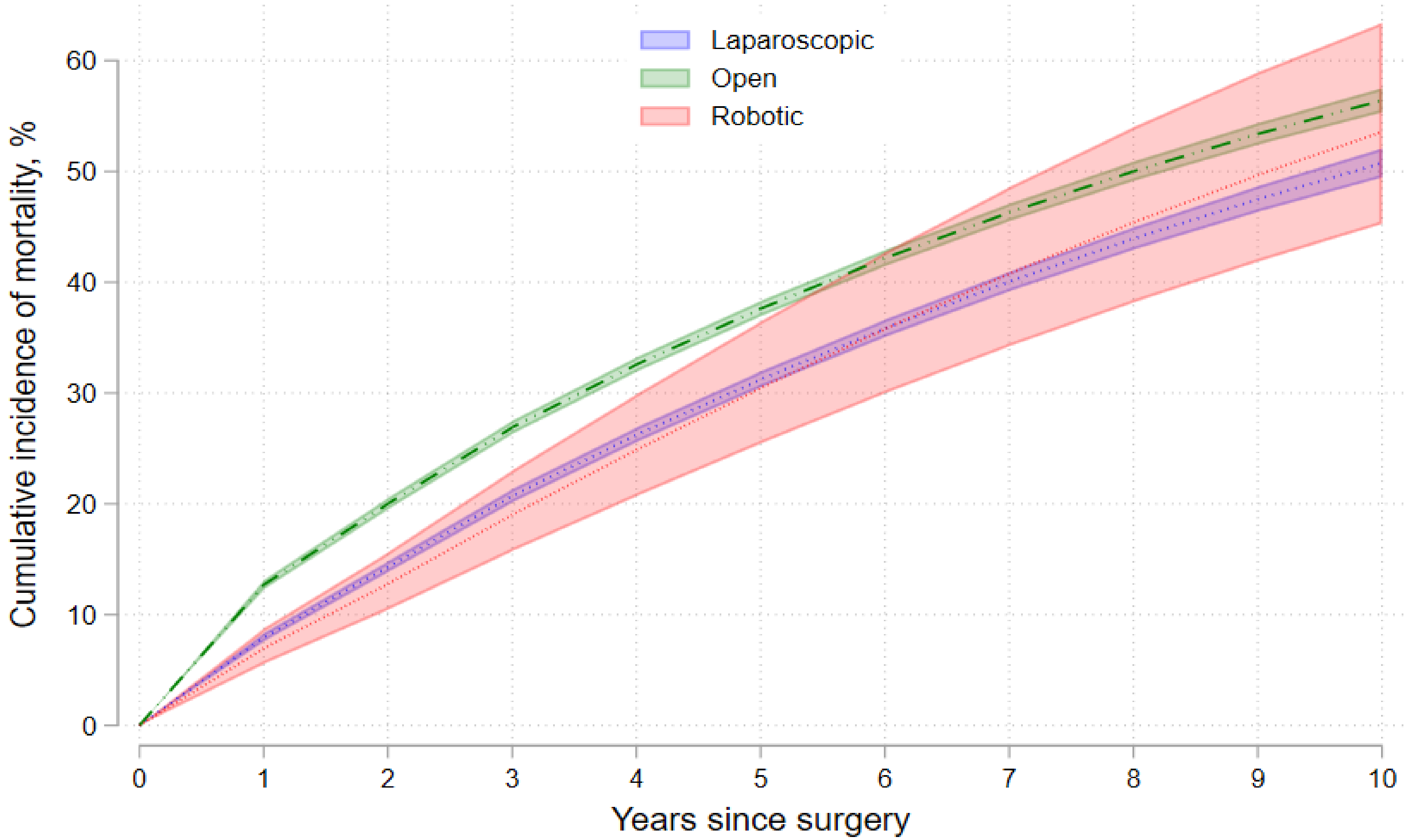

Supplemental Figure 4: Cumulative incidence of mortality for stage 3 colon cancer.

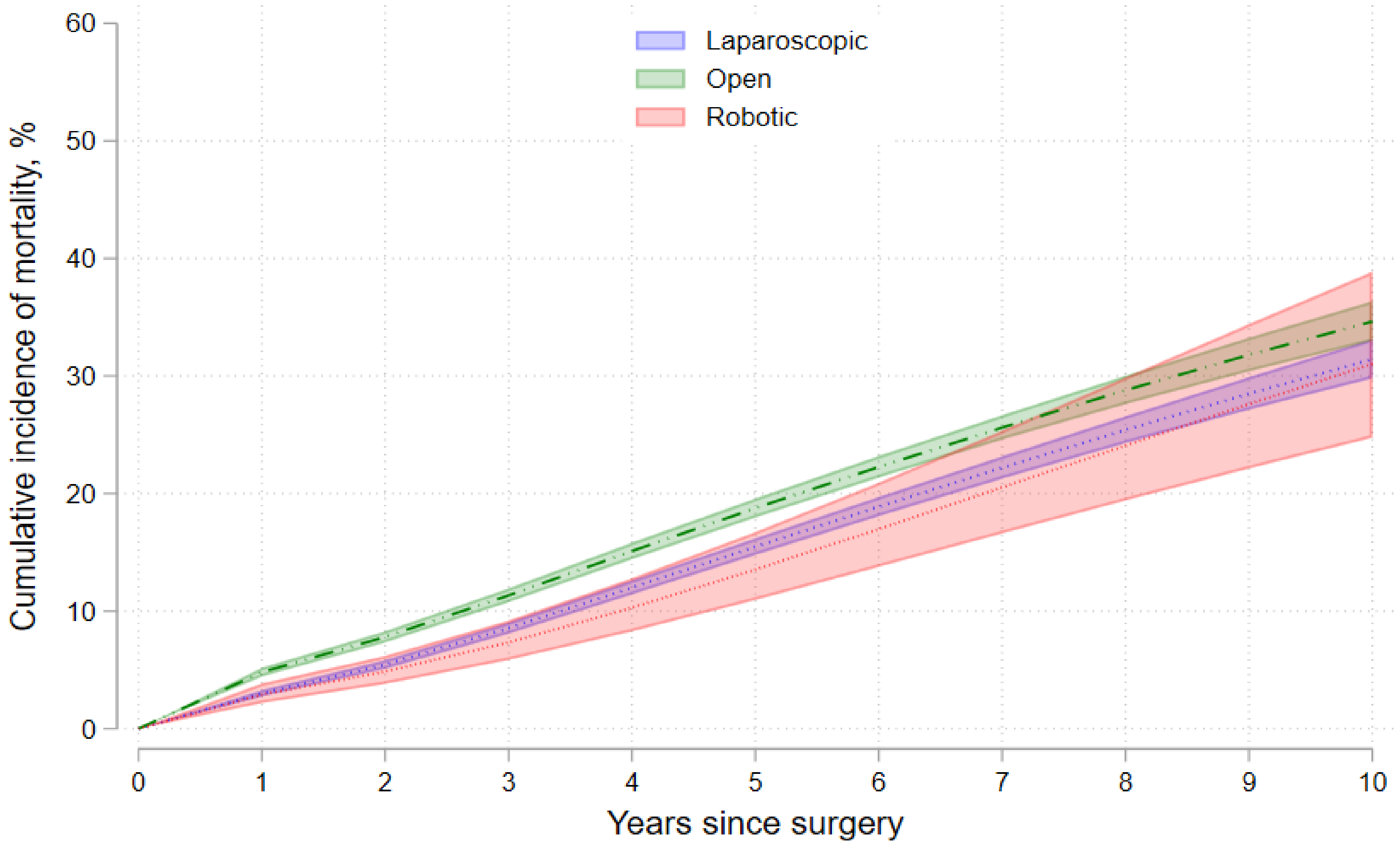

Supplemental Figure 5: Cumulative incidence of mortality for stage 1 rectal cancer.

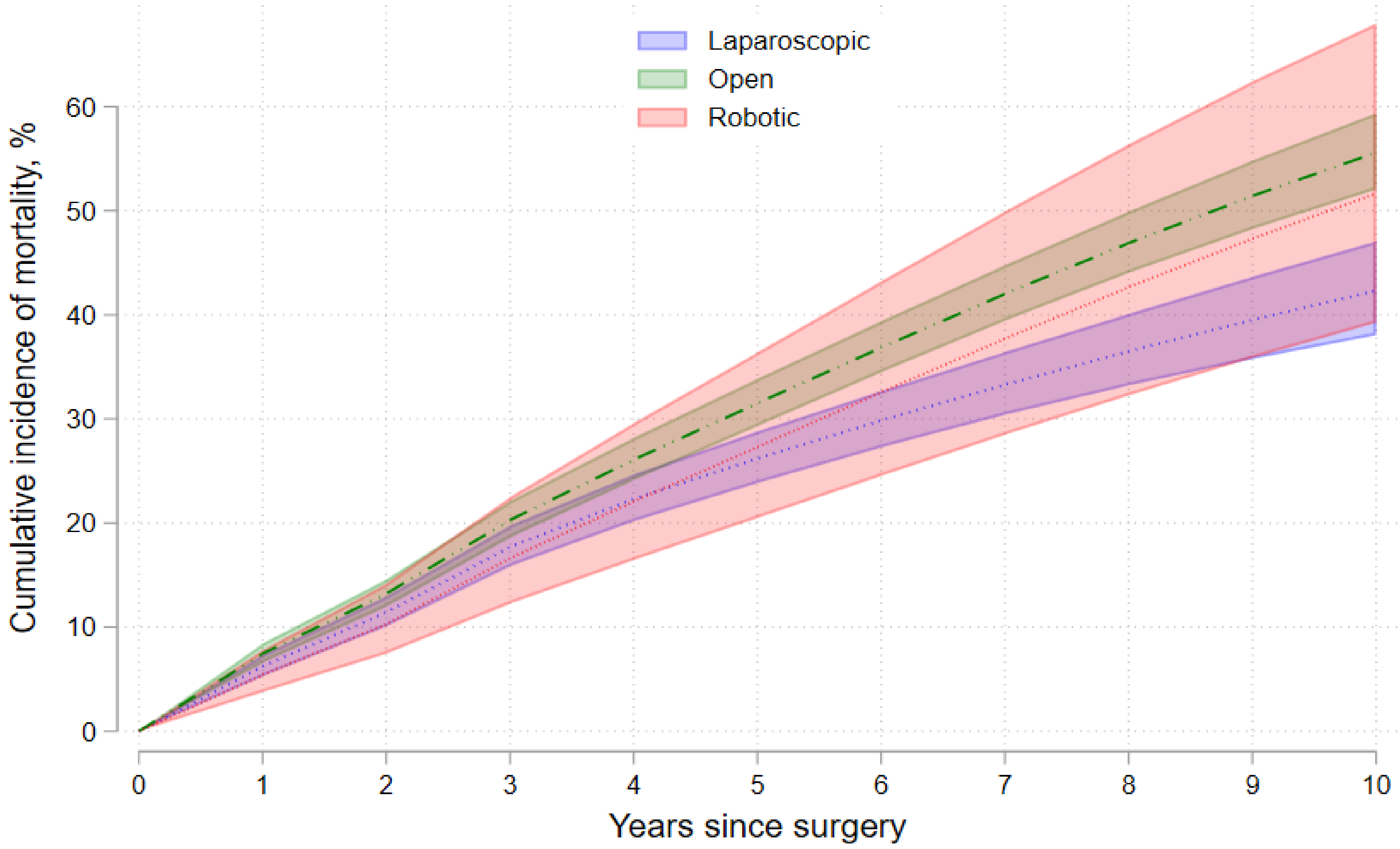

Supplemental Figure 6: Cumulative incidence of mortality for stage 3 rectal cancer.
